# Microsurgery for vestibular schwannoma: analysis of short-term clinical outcome

**DOI:** 10.1186/s41016-022-00306-z

**Published:** 2022-12-21

**Authors:** Xu Wang, Mingchu Li, Xinru Xiao, Ge Chen, Jie Tang, Qingtang Lin, Hongchuan Guo, Gang Song, Xiaolong Wu, Yuhai Bao, Jiantao Liang

**Affiliations:** 1 Department of Neurosurgery, Xuan Wu Hospital of Capital Medical University, Beijing, China; 2 China International Neuroscience of Institute (CHINA-INI), Beijing, China

**Keywords:** Vestibular schwannoma, Inner auditory canal, Retro-sigmoid approach, Facial paralysis

## Abstract

**Background:**

Total removal of the vestibular schwannoma when preserving the function of the facial nerve is difficult. The objective of the current study was to investigate the short-term clinical outcome of vestibular schwannoma removal via retro-sigmoid approach.

**Methods:**

One-hundred consecutive patients diagnosed with vestibular schwannoma were surgically treated between December 2018 and August 2019 in Xuanwu Hospital, Capital Medical University. The clinical classification, surgical position, gross total removal rate, the anatomical and functional preservation rates of facial nerve, and the postoperative complications were retrospectively analyzed.

**Results:**

All 100 patients including 34 males and 66 females were operated on via retro-sigmoid approach. According to Koos vestibular schwannoma grading system, 18 cases were grade 2, 34 cases were grade 3, and 48 cases were grade 4. According to Hannover vestibular schwannoma grading system, 5 cases were T2, 6 cases were T3a, 8 cases were T3b, 30 cases were T4a, and 51 cases were T4b. Seventy-three surgeries were performed under lateral position, and 27 cases were operated under semi-sitting position. The gross total removal rate was 90.0%; the anatomic reservation rate of the facial nerve was 96.0%. According to the House-Brackman system, the facial nerve function was grades 1–2 in 78.0% cases, grade 3 in 7.0% cases, and grades 4–5 in 15% cases. For patients with effective hearing before operation, the hearing reservation rate was 19.0%. Two patients (2.0%) developed intracranial hematoma after operation.

**Conclusion:**

Most vestibular schwannoma could be completely removed with good postoperative facial nerve function. If total removal of tumor is difficult, we should give priority to the functional preservation of the nerve function.

## Background

Vestibular schwannoma (VS) was firstly described by a Dutch anatomist in 1777. In 1894, Charles Balance successfully performed the first VS surgery. The morbidity and mortality rates after VS surgery were high in the early stage. As a benign intracranial tumor, total removal of the VS is generally recognized as the best treatment. During the last several decades, the surgical results and functional preservation rate of facial nerve have got significant improvement, whereas total resection of the tumor at the time of well preservation of facial nerve and cochlear nerve is still a great challenge. Many different surgical techniques for removal of tumor and preservation of facial nerve were proposed. Nowadays, protection of the hearing function, avoiding of postoperative complications, and postoperative rehabilitation are main objectives [1–4]. In the current study, we retrospectively analyzed the clinical and radiological data of 100 patients diagnosed with VS, and then the surgical details of preservation of the facial nerve were discussed.

## Methods

### Patients

**Fig. 1:**
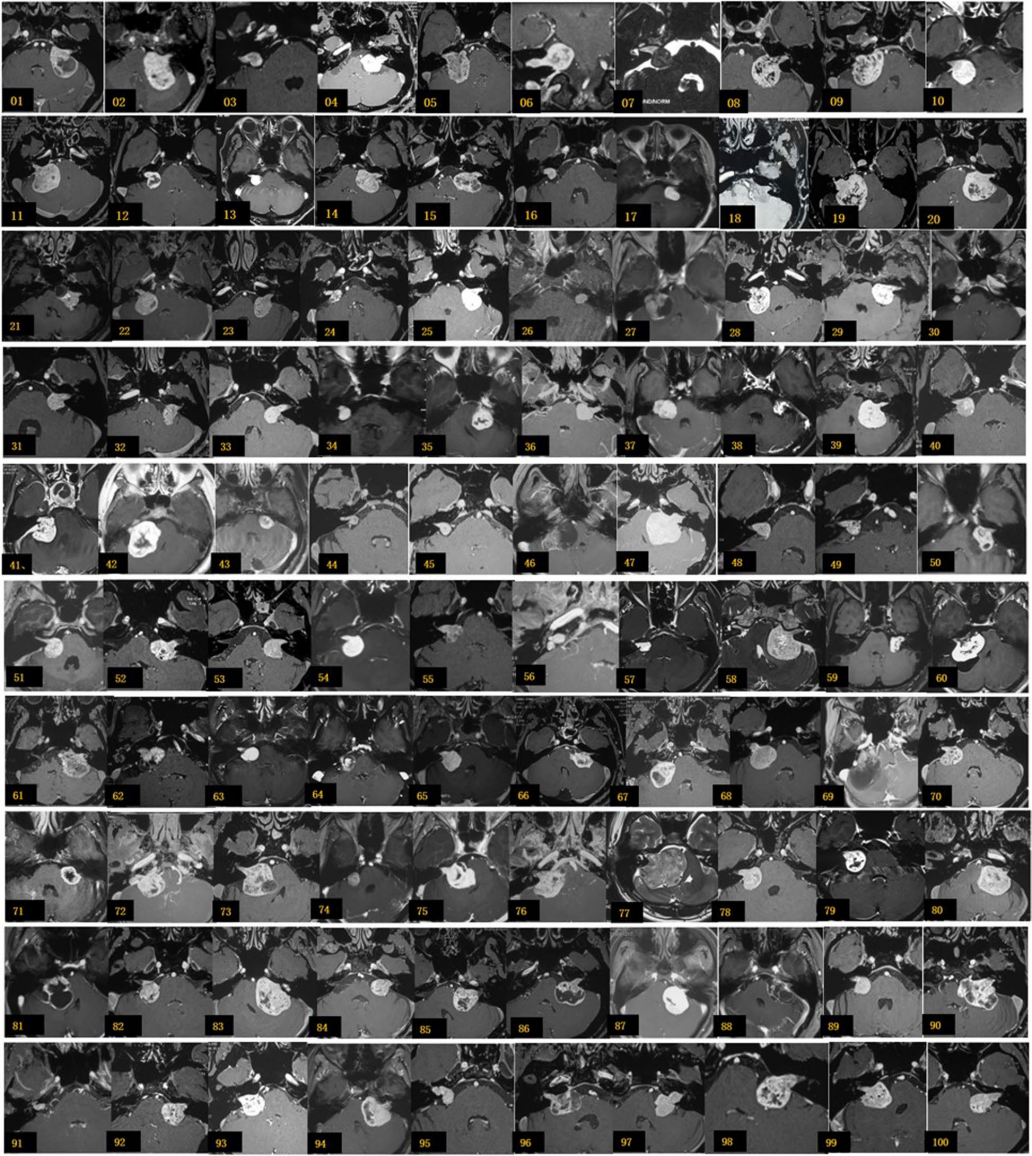
Preoperative brain MRI of the 100 cases

Between September 2018 and August 2019, 106 VS resection surgery was performed at Xuanwu Hospital of Capital Medical University. All admitted patients were aged from 20 to 75 years old, with American Society of Anesthesiologists grades 1–3, and diagnosed with VS based on brain MRI. Patients diagnosed with neurofibromatosis-2 and those who had received Gamma Knife or surgical treatment were excluded. As a result, 100 cases were included in the current study. The tumor classification, operative position, total removal rate of tumor, preservation rate of facial and cochlear nerves, and postoperative complications were retrospectively analyzed. Preoperative brain MRI of the 100 cases was shown in Fig. 1.

### Surgical technique and details

**Fig. 2:**
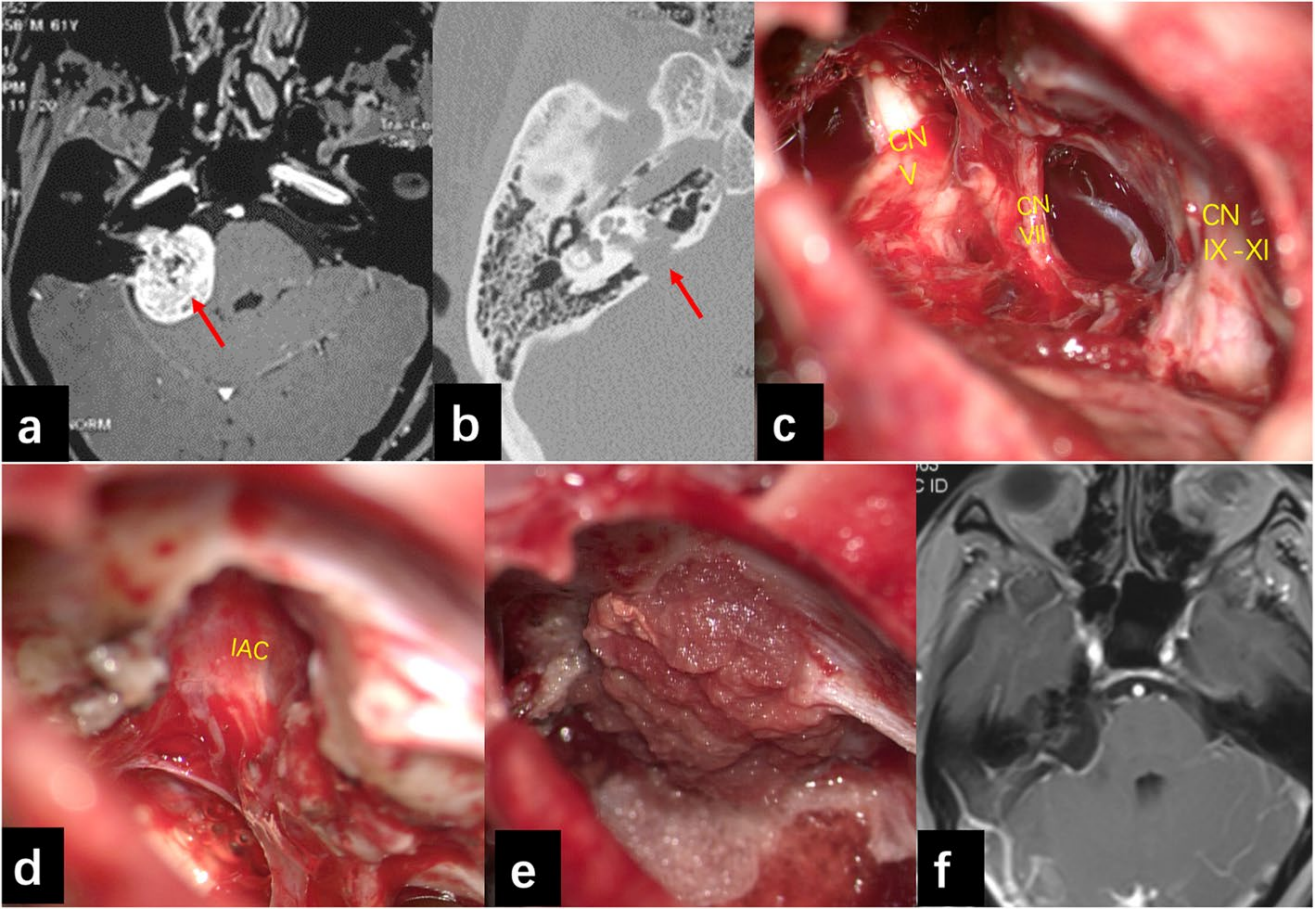
Radiological data and intraoperative photo of right VS. **a** Preoperative brain MRI showed large VS in the right side which compressed the brainstem. **b** Preoperative bone window CT scan showed the enlarged IAC. **c** The trigeminal nerve, facial nerve, and lower nerves were well preserved after total removal of the VS. **d** Facial nerve inside the IAC could be seen after drilling of the posterior wall of IAC and removal of the tumor inside. **e** The IAC was repaired by some muscular tissue after operation. **f** Brain MRI rechecked after operation showed the tumor was totally removed

The intraoperative electrophysiological monitoring was routinely adopted, including somatosensory-evoked potential, motor-evoked potential, monitoring of the trigeminal nerve, the facial nerve, and the lower nerves. For patients with available hearing before operation, brainstem auditory-evoked potential was also monitored. All patients were operated via retro-sigmoid approach, with lateral position used in 73 cases, and semi-sitting position was adopted in another 27 cases. During the operation performed under semi-sitting position, end-tidal carbon dioxide, transesophageal echocardiography, and precordial Doppler were used for the monitoring of intraoperative venous air embolism. The tumor could be exposed after opening of the dura and retraction of the cerebellum. The general location of facial nerve was estimated firstly with the help of neurophysiological monitoring. After debulking of the tumor, posterior wall of the internal auditory canal (IAC) was carefully drilled. The bony structure of the IAC that needs to be drilled away was based on its depth, tumor volume inside the IAC, and the preoperative hearing condition. The tumor inside the IAC should be removed under strict neurophysiological monitoring. Then, the main direction of the facial nerve running in the cerebellopontine angle could be confirmed in most patients. The membrane structure on the surface of the tumor should be preserved when dissecting the tumor adhered to the brainstem, cerebellum, and the cranial nerves. After total removal of the tumor, some muscular tissue and medical glue were used for the repair of posterior wall of the IAC to prevent for the postoperative cerebrospinal fluid (CSF) leakage (Fig. 2).

### Statistical analysis

Continuous variables were presented as the mean ± standard deviation, and categorical variables were presented as percentages. All statistical analyses were performed by the SPSS software (Version 27.0, IBM Corporation, Armonk, New York, USA). *P* < 0.05 was considered statistically significant.

## Results

The 100 patients included 34 males and 66 females with age ranging from 22 to 72 years. Ten cases were in their 20s, 15 cases were in the 30s, 28 cases were in the 40s, 31 cases were in the 50s, 15 cases were in the 60s, and 1 case was in his 70s. The VS was classified according to the Koos system and Hannover system (Table 1). The clinical manifestation before surgery was shown in Table 2.

**Table 1:** Baseline information of the patients

Number of patients	100
Mean age	48.8 (22–72)
Age distribution (years)	Number
21–29	10 (10%)
30–39	15 (15%)
40–49	28 (28%)
50–59	31 (31%)
60–69	15 (15%)
70–79	1 (1%)
Gender ratio (female: male)	66:34
Tumor side left/right	46:54
Koos grading	Number
Grade 1	0
Grade 2	18 (18%)
Grade 3	34 (34%)
Grade 4	48 (48%)
Hannover grading	Number
T1	1 (1%)
T2	5 (5%)
T3a	6 (6%)
T3b	8 (8%)
T4a	30 (30%)
T4b	50 (50%)
Tumor characteristics	Number
Cystic	30 (30%)
Solid	70 (70%)

**Table 2:** Clinical manifestation before surgery

Preoperative symptom	Number of patients
Tinnitus	39 (39.0%)
Hearing decrease	64 (64.0%)
Unsteady walking	4 (4.0%)
Dizziness	12 (12.0%)
Trigeminal neuralgia	3 (3.0%)
Increased intracranial pressure	5 (5.0%)
Without symptom	6 (6.0%)

**Fig. 3:**
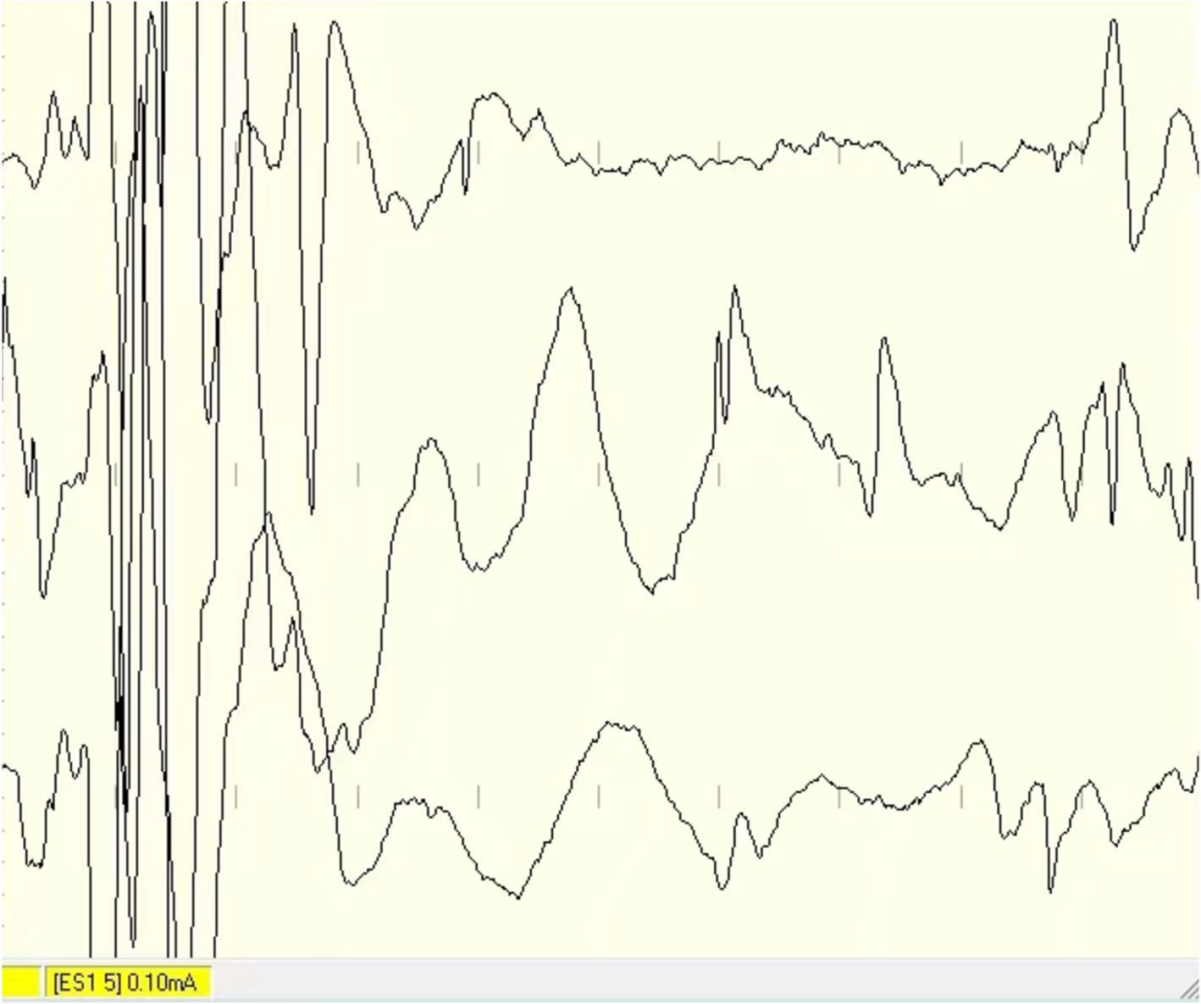
Intraoperative photograph. Intraoperative electrophysiological monitoring showed active electrical response of the facial nerve when the VS was near totally removed

Seventy-three patients were operated on with lateral position, and 27 patients were operated on with semi-sitting position. The gross-total removal rate, near-total removal rate, and subtotal removal rate were 90.0%, 6.0%, and 4.0%, respectively. In 96.0% of the cases, the facial nerve was anatomically preserved. At the discharge, 78 patients got a good facial nerve outcome (H-B grades 1–2 78%), 7 patients got fair facial nerve outcome (H-B grade 3 7%), and 15 patients got poor facial nerve outcome (H-B grades 4–5, 15%). The poor facial nerve outcome rate of patients with Koos grade 4 VS was insignificantly higher than that of patients with Koos grades 1–3 VS (20.8% vs. 9.6%, *P* = 0.12). Koos grade 4 was the only independent predictor for the poor facial nerve outcome after surgery. Among the 44 patients with feasible hearing before operation, the hearing was preserved in 10 (22.7%) cases after operation. The CSF leakage developed in 1 case (1.0%) after operation. Intracranial hematoma occurred in 2 patients (2%), who achieved a fair outcome at discharge after conservative therapy (Table 3). If the facial nerve was severely compressed and closely adhered to the tumor, small part of tumor was remained, and the facial nerve should be carefully protected based on the electrophysiological monitoring results (Fig. 3).

**Table 3:** Surgical outcome

Extent of resection	Number
Gross total removal	90 (90%)
Near-total removal	4 (4%)
Subtotal removal	6 (6%)
Postoperative complications	Number
CSF leakage	1 (1%)
Hematoma	2 (2%)
Facial nerve function	
Grade 1	62 (62%)
Grade 2	16 (16%)
Grade 3	7 (7%)
Grades 4–5	15 (15%)

## Discussion

### Surgical strategy for giant VS

VS is common intracranial tumor, which could be cured by total resection. The tumor should be removed as much as possible to prevent for the recurrence. On the other hand, the function of facial nerve is also very important for the quality of life. Total removal of giant VS is achievable at the technical level, but severe injury of facial nerve is sometimes inevitable even for those most experienced surgeons [5]. For instance, if the facial nerve is severely compressed and closely adhered to the tumor, intact dissection of the facial nerve from the tumor is extremely difficult. When total removal of VS is contradictory to the preservation of the facial nerve, most doctors give preference to the preservation of facial nerve. In the current study, the anatomic reservation rate of the facial nerve was 96.0%. According to Madjid Samii et al., the anatomic reservation rate of the facial nerve for T4 grade VS was 97% [6, 7]. As a result, more effort will be needed for the protection of facial nerve in the future.

### Definition of the resection extent of the VS

The resection extent of the VS is usually based on the subjective judgement of the surgeon during the operation and the diameter or volume of the residual tumor shown on the rechecked MRI after operation [8]. In the current study, we adopted both the intraoperative finding and postoperative MRI results for the evaluation of the resection extent. Gross total removal (GTR) was defined by total resection of tumor under microscope and confirmed by postoperative brain MRI that no tumor remained. Near-total removal (NTR) meant that a thin section of tumor was remained during operation which could not be seen on the postoperative brain MRI. Subtotal removal (STR) meant that small part of tumor was remained during operation which could be seen on the postoperative brain MRI.

### Indications for NTR or STR of giant VS

NTR or STR of VS should be considered in some situations. Firstly, if the facial outcome after dissection from the tumor could not be precisely and objectively evaluated during the surgery, NTR or STR may be sensible, because we could not identify the “critical point” that the facial nerve was permanently injured [9]. On the other hand, the remained tumor was often stable or even shrink during the follow-up and could be effectively controlled by radiotherapy [10].

If the tumor was closely related to the facial nerve, brainstem, or vascular structures that dissection of the facial nerve was quite difficult or even impossible, total removal of the giant VS was not recommended. In some patients who were elderly or accompanied with other severe complications, whose hearing of the contralateral side was lost, or unexpected hemorrhage was intraoperatively encountered, total removal was also unadvisable [11, 12].

In the current study, total removal was not achieved in 10 cases. In two elderly patients who were accompanied with other diseases, the objectives of surgery were to relieve the brainstem compression and the severe trigeminal nerve due to tumor compression, so NTR and STR of tumor were performed. In one patient with evident brainstem edema, a thin layer of tumor adhered to the brainstem was remained during the operation. In one patient, the facial nerve is located right on the back of tumor; exposure of the ventral tumor was very difficult, so the total removal was not achieved. In another 6 cases, the tumor was closely related to the facial nerve; small part of tumor on the surface of facial nerve was remained. The postoperative brain MRI showed thin slice of tumor was remained in 6 patients (STR); in another 4 cases, the postoperative brain MRI could not reveal the residual tumor (NTR).

### The postoperative treatment of the residual VS

The prognosis of residual VS including the tumor regrowth time, regrowth velocity, and whether need reoperation or other treatment has been reported in many researches. The recurrence rate is higher in patients who received STR or NTR, compared with those total removal was achieved. What’s more, the recurrence rate increased as the follow-up period extended. Carlson et al. reported that the recurrence rate was 22% after a follow-up of 3.5 years [12]. Chen et al. reported the recurrence rate was 18% after 3.8 years of follow-up [8]. Seol et al. reported that after a mean follow-up of 4.6 years, the residual VS regrew in 28% of patients [13], whereas there is also research reported that the VS could shrink in the early period after operation [14]. Therefore, patients who received STR of VS should be closely followed after operation.

Many scholars suggest that radiotherapy should be conventionally arranged early after operation if the VS was not totally removed [15]. Some others recommend that the radiotherapy should be performed only when regrowth of the residual VS was confirmed [16]. In the current study, STR or NTR of the VS was performed in 10 patients, in view of the small volume of the residual tumor; none of them received radiotherapy. The treatment strategy will be depended on the growth tendency of the tumor during the close follow-up.

## Conclusion

Most giant VS could be completely and safely removed with good postoperative facial nerve function. If total removal is difficult, functional preservation of the facial nerve function should be firstly considered.

## Data Availability

The datasets used and/or analyzed during the current study are available from the corresponding author on reasonable request.

## Abbreviations

VS: Vestibular schwannoma
IAC: Internal auditory canal
CSF: Postoperative cerebrospinal fluid
GTR: Gross total removal
NTR: Near-total removal
STR: Subtotal removal

## References

[1] Landry AP, Yang K, Wang JZ, Gao AF, Zadeh G (2022). Outcomes in vestibular schwannoma treated with primary microsurgery: clinical landscape. J Clin Neurosci.

[2] Martinez-Perez R, Ung TH, Samy A, Youssef. (2021). The 100 most-cited articles on vestibular schwannoma: historical perspectives, current limitations, and future research directions. Neurosurg Rev.

[3] Schackert G, Susann Ralle K, Martin D, Reiss G, Kowalski M, Sobottka SB (2021). Vestibular schwannoma surgery: outcome and complications in lateral decubitus position versus semi-sitting position-a personal learning curve in a series of 544 cases over 3 decades. World Neurosurg.

[4] Jia H, Nguyen Y, De Seta D, Hochet B, Smail M, Bernardeschi D (2020). Management of sporadic vestibular schwannoma with contralateral nonserviceable hearing. Laryngoscope..

[5] Zou P, Zhao L, Chen P, Xu H, Liu N, Zhao P (2014). Functional outcome and postoperative complications after the microsurgical removal of large vestibular schwannomas via the retrosigmoid approach: a meta-analysis. Neurosurg Rev.

[6] Samii M, Gerganov VM, Samii A (2010). Functional outcome after complete surgical removal of giant vestibular schwannomas. J Neurosurg.

[7] Gharabaghi A, Samii A, Koerbel A, Rosahl SK, Tatagiba M, Samii M (2007). Preservation of function in vestibular schwannoma surgery. Neurosurgery..

[8] Chen Z, Prasad S, Di Lella F, Medina M, Piccirillo E, Taibah A (2014). The behavior of residual tumors and facial nerve outcomes after incomplete excision of vestibular schwannomas. J Neurosurg.

[9] Anaizi A, Gantwerker E, Pensak M, Theodosopoulos P (2014). Facial nerve preservation surgery for koos grade 3 and 4 vestibular schwannomas. Neurosurgery..

[10] Tomita Y, Tosaka M, Aihara M, Horiguchi K, Yoshimoto Y (2015). Growth of primary and remnant vestibular schwannomas: a three-year follow-up study. World Neurosurg.

[11] Carlson M, Habermann E, Wagie A, Driscoll C, Van Gompel J, Jacob J (2015). The changing landscape of vestibular schwannoma management in the United States—a shift toward conservatism. Otolaryngol Head Neck Surg.

[12] Carlson M, Van Abel K, Driscoll C, Neff B, Beatty C, Lane J (2012). Magnetic resonance imaging surveillance following vestibular schwannoma resection. Laryngoscope.

[13] Seol H, Kim C, Park C, Kim C, Kim D, Chung Y (2006). Optimal extent of resection in vestibular schwannoma surgery: relationship to recurrence and facial nerve preservation. Neurol Med Chir (Tokyo).

[14] Akinduro O, Lundy L, Quinones-Hinojosa A, Lu V, Trifiletti D, Gupta V (2019). Outcomes of large vestibular schwannomas following subtotal resection: early post-operative volume regression and facial nerve function. J Neuro-Oncol.

[15] Van de Langenberg R, Hanssens P, van Overbeeke J, Verheul J, Nelemans P, de Bondt B (2011). Management of large vestibular schwannoma. Part I. planned subtotal resection followed by gamma knife surgery: radiological and clinical aspects. J Neurosurg.

[16] Zumofen D, Guffi T, Epple C, Westermann B, Krähenbühl A, Zabka S (2017). Intended near-total removal of Koos grade IV vestibular schwannomas: reconsidering the treatment paradigm. Neurosurgery.

